# Burden of hypertension and type 2 diabetes in Tamale Metropolis: a case study of Tamale teaching hospital

**DOI:** 10.1186/s12889-025-23979-4

**Published:** 2025-08-26

**Authors:** Ishamatu Mohammed Yakubu, Mustapha Alhassan, Ebenezer Tawiah Arhin, Hudu Mohammed

**Affiliations:** 1 Development of Education Directorate, Tamale Technical University, Tamale, Ghana; 2 Department of Health Sciences Education, University for Development Studies, Tamale, Ghana; 3 Department of Statistical Sciences, Tamale Technical University, Tamale, Ghana; 4 Department of Mathematical Sciences, Kumasi Technical University, Kumasi, Ghana

**Keywords:** Hypertension, Type 2 diabetes, Prevalence

## Abstract

Hypertension and type 2 diabetes mellitus are a pair of prevalent chronic non-communicable ailments that present significant obstacles to the global well-being of the public. The study investigates the burden of Hypertension and Type 2 Diabetes in Tamale metropolis. The target population used for the study was all outpatients from the Tamale Teaching Hospital. Secondary source of data was employed for the study. The findings reveal significant variations in hypertension prevalence across demographic categories, with the highest rates found in the 55–64 age group at 31.4% (95% CI: 26.3%—36.5%) and all age groups demonstrating statistical significance (*p* < 0.0001). Findings further show the prevalence of Type 2 diabetes peaks at 28.8% in the 65–74 age group, while the 25–34 age range has the lowest rate at 1.5%. Findings from the regression analysis reveal that gender and patients’ place of residence significantly influence type 2 diabetes, while age is the only variable that shows a statistically significant association with hypertension. The study concluded that Type 2 diabetes and hypertension is prevalent among older age, higher weight, male gender, self-employed, and lower education. The study recommends for implementation of targeted screening programs focusing on older adults, especially females, and those in self-employed, for Type 2 diabetes and Hypertension.

## Introduction

Hypertension and type 2 diabetes mellitus are a pair of prevalent chronic non-communicable ailments that present significant obstacles to the global well-being of the public. Hypertension, which is defined by high blood pressure, and type 2 diabetes, a metabolic disease caused by lack of insulin and hyperglycemia, frequently coexist and work together to increase the risk of cardiovascular events, kidney damage, and death [8, 71]. According to Chobanian et al. [21] and Kasper (2015), hypertension is projected to be the cause of 7.5 million deaths annually worldwide, accounting for a significant portion of the world's sickness burden. The Global Health Observatory Report estimates that in 2008, around 40% of persons 25 years of age and older had hypertension overall (Institute of Medicine Committee, 2010). 972 million persons worldwide were predicted to have hypertension in 2000; 333 million lived in economically developed nations and 639 million in economically developing nations [51]. It is anticipated that 1.56 billion persons worldwide would have hypertension by 2025 [51]

Approximately 1.13 billion people worldwide suffer with hypertension, and the incidence of this condition is on the rise, especially in low- and middle-income countries, according to the World Health Organization (*WHO*, 2023). Similarly, type 2 diabetes is predicted to affect 463 million people worldwide by 2030, contributing to an epidemic-like situation (Magliano et al., 2021). In earlier times, the area was closely tied to infectious ailments; nonetheless, transitions in disease patterns, urbanization, lifestyle shifts, and an older demographic have led to a surge in non-communicable disease (NCD) occurrences [60].

With prevalence rates showing significant variation among countries, hypertension in particular is a growing concern throughout the African continent. According to a thorough analysis, the prevalence of hypertension in Africa is roughly 30%, which is much higher than the global average [64]. According to Zeru et al. [90], non-infectious diseases, particularly diabetes mellitus (DM) and hypertension, have alarmingly increased in Ethiopia. According to a meta-analysis carried out in Ethiopia, the prevalence of hypertension was 20.63% and the prevalence of type 2 diabetes was 6.5% [80]. In Ethiopia, the challenges associated with diabetes and the associated heart-related issues, such as hypertension, nerve problems, kidney problems, coronary artery disease, and strokes, have become significantly more urgent [33].

Due in large part to a lack of adequate medical facilities, limited access to essential medications, and challenges with disease monitoring and management, high blood pressure and type 2 diabetes are prevalent on the African continent. Since the healthcare systems in many African nations are not prepared to handle the rising demand for NCD care, there are significant gaps in prevention, diagnosis, and treatment services [63]. Moreover, variations in the prevalence of type 2 diabetes and hypertension among different African populations are caused by differences in economic standing and the availability of medical resources. According to [60], vulnerable groups—such as those who are poor, live in rural areas, or reside in informal settlements—often encounter obstacles when trying to access healthcare facilities. They also run a higher risk of having their NCDs misdiagnosed or inadequately addressed.

Even though the health risks associated with hypertension and type 2 diabetes are becoming more widely recognized, less is known about the epidemiological patterns, risk factors, and management issues associated with these conditions in sub-Saharan African cities like Tamale Metropolis in Ghana. Existing literature primarily focuses on high-income countries and broader regional analyses, overlooking the unique contextual factors influencing the burden of these diseases in low-resource settings. Furthermore, little thorough study has been done on the intersections of type 2 diabetes and hypertension, especially when it comes to co-morbidity and related consequences. Understanding the interplay between these conditions is crucial for developing holistic intervention strategies tailored to the complex healthcare needs of affected populations. Thus, in an effort to close the research gap and provide targeted public health interventions and locally-specific healthcare delivery techniques, this study examines the prevalence of type 2 diabetes and hypertension in the Tamale Metropolis.

## Materials and methods

### Study area

The Tamale Metropolis was the study area of the research. The Northern Region is home to 26 districts, one of which being Tamale Metropolis. The Sagnarigu District and Savelugu Municipality form its northern and western borders, while the Mion District and the East Gonja District form its eastern and southern, and the Central Gonja District forms its southwestern, neighboring ones. Its location is in the very heart of the area. The land area of the Metropolis is around 646,90,180 square kilometers (*GSS, 2021*). In terms of latitude and longitude, the Metropolis is situated between 9º16 and 9º 34 north and 0º 36 and 0º 57 west.

**Fig. 1:**
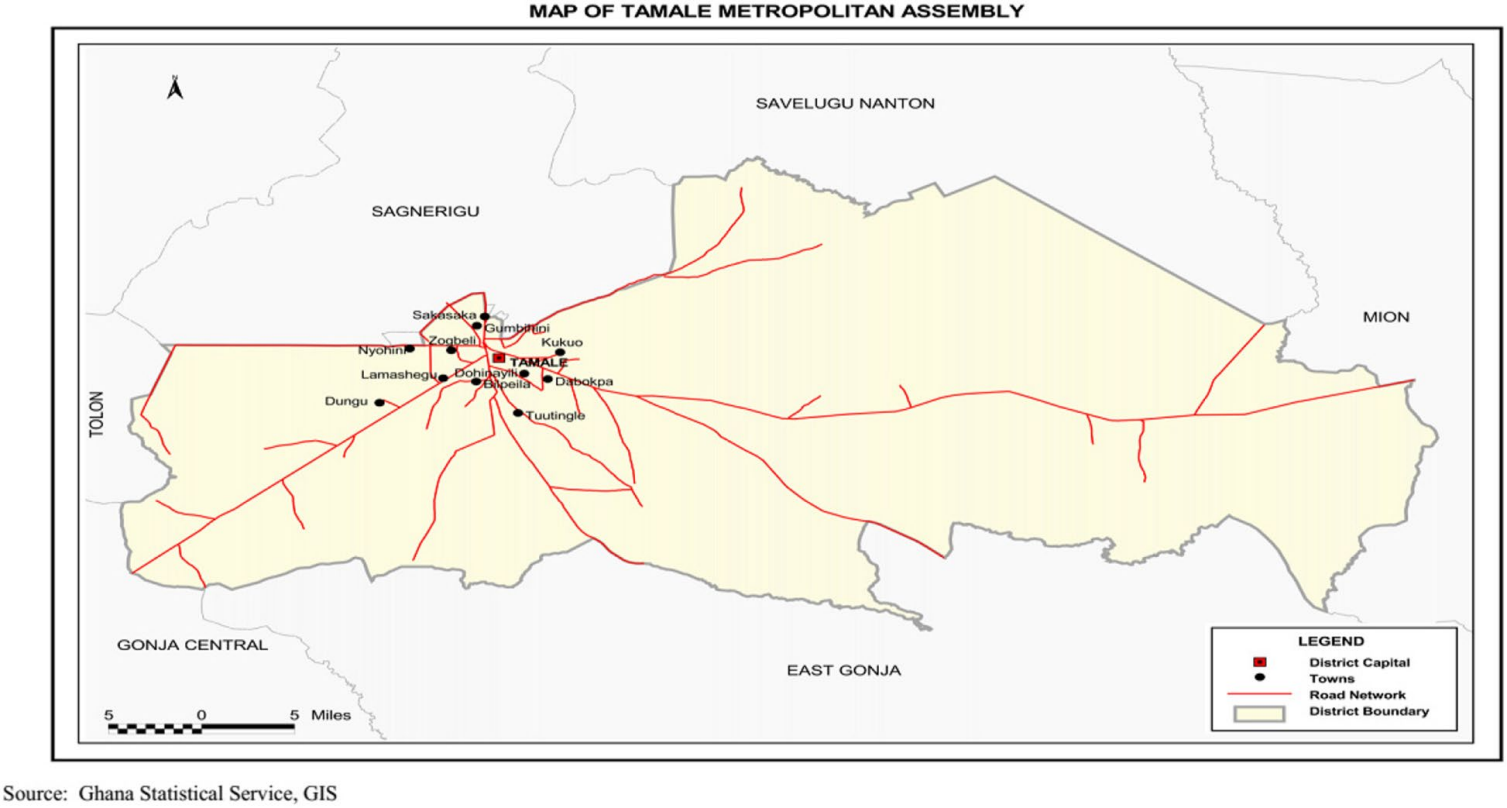
The map of Tamale metropolitan assembly

A total of 374,744 people live in the Metropolis, including 189,693 women and 185,051 men (50.6% and 49.4%, respectively). The populations of the region and the country, respectively, are roughly 16.2% and 1.2% (*GSS, 2021*). With 89,011 households and an overall household size of 4.1 people, the density of population is 825 per sq km, which is lower than the area average of 5.2. Around 27.2% of people can read and write exclusively in English, 9.4% can read and write in Ghanaian, and 61.8% can read and write in both English and Ghanaian. The population has 0.4% literate in English and 0.4% in French, for a total literacy rate of 1.1% for the three languages (English, French, and a Ghanaian language). There are more female illiterates than male illiterates. The map of Tamale Metropolitan Assembly is shown in Fig. 1 below.

### Target population

The target population for the study comprised all outpatients at Tamale Teaching Hospital.

### Study population

The study population consisted of outpatients diagnosed with type 2 diabetes and hypertension, as documented in the outpatient morbidity register of the Tamale Teaching Hospital. Cases were chosen via a past review of secondary data from the hospital's Health Information System (HIS), encompassing records from January 2023 to December 2023. Only patients with a verified diagnosis of one or both diseases, as recorded by a healthcare professional at an outpatient consultation, were incorporated into the analysis. A total of 205 qualified cases that satisfied these inclusion criteria were extracted for the study. Patients with incomplete records or ambiguous diagnoses were eliminated to guarantee data precision and uniformity.

### Source of data

The information was obtained from a secondary source. Data regarding type 2 diabetes and hypertension cases were obtained from the Health Information System (HIS) of the Tamale Teaching Hospital since it serves as the reference center within the northern sector of Ghana.

### Study variables

For the study, two sets of variables were chosen. These are both independent and dependent variables. The glucose level of people with type 2 diabetes and their blood pressure served as the dependent variables. The socio-economic characteristics (level of education, age, occupation, weight, place of residence, and gender) of patients were the independent variables for this study.

### Data analysis

The data was analyzed using both descriptive and inferential statistical techniques. Descriptive statistics include the cross-tabulation and the percentages. An inferential technique called multivariate multiple regression was used to model the socio-economic traits linked with type 2 diabetes and hypertension.

### Model specification

The Multivariate Multiple Regression model is a suitable analytical tool in cases where one finds two dependent variables together with several independent variables. This particular regression method clarifies the variability of several dependent variables by means of a set of independent variables and allows their simultaneous modeling, therefore recognizing their interrelationships.

### Model Structure

The multivariate multiple regression model can be written as:

$$Y=X\beta +\epsilon$$

where:

Y is an n × m matrix of dependent variables, with n observations and mm dependent variables.

X is an n × p matrix of independent variables, with p predictors.

$\beta$ is a p × m matrix of coefficients.

$\epsilon$ is an n × m matrix of errors or residuals.

## Estimation of coefficient

Coefficient B can be estimated using multivariate ordinary least squares (OLS), which minimizes the sum of squared residuals. This is often done using matrix algebra:

$$\beta ={\left({X}^{T}X\right)}^{-1}{X}^{T}Y$$

### Assumptions of multivariate multiple regression

The Multivariate Multiple Regression model bases its principal assumptions on the following premises regarding the underlying data:

i. The dependent variables is continuous.
ii. Linearity
iii. Independence: The observations should be independent of each other, implying that the residuals (errors) for one observation are not correlated with the residuals of another.
iv. Homoscedasticity: The residuals (errors) should have constant variance across all levels of the independent variables, meaning that the spread of the residuals should be roughly the same at all points along the range of the predictors.
v. Normality: The residuals (errors) should be approximately normally distributed, particularly important for hypothesis testing and constructing confidence intervals.

### The 95% confidence limits for prevalence of Type 2 diabetes and Hypertension disease

The 95% confidence limits for the prevalence of disease are a statistical tool used to determine the range of values within which the true population prevalence is expected to fall with 95% probability based on a particular sample[16].

In order to determine the 95% confidence limits for illness prevalence, the formula below is used:

$$\text{Lower limit}=\left(\frac{n}{N}\right)-\left(1.96\right)\sqrt{\frac{\left(\frac{n}{N}\right)\left(1-\left(\frac{n}{N}\right)\right)}{N}}$$

$$\text{Upper limit}=\left(\frac{n}{N}\right)-\left(1.96\right)\sqrt{\frac{\left(\frac{n}{N}\right)\left(1-\frac{n}{N}\right)}{N}}$$

where:

$$\frac{n}{N}=\text{proportion of individuals in the population with the disease}$$

The value of the z-score for a 95% confidence interval = 1.96.

The total number of people in the population (*N*).

The number of patients in the population (*n*).

### Ethical considerations

This study utilized secondary data obtained from Tamale Teaching Hospital and did not involve the prospective recruitment of human participants by the researchers. Data were accessed for the research from 10th June, 2024 to 10th November, 2024. The data were not publicly available but were accessed with the appropriate institutional permissions. All data collected during the study were kept confidential and only accessible to the research team. Participants'names and other identifying information were not included in any reports or publications resulting from the study. Ethical approval was obtained from the University for Development Studies Institutional Review Board (UDSIRB) before the study was conducted.

## Results

The demographic features of patients with type 2 diabetes are presented in Table 1. The age distribution reveals a substantial concentration in older age groups, with the most of the patients (28.8%) being within the age range of 65–74 years old. There is a larger prevalence of type 2 diabetes among females, with 76.1% of patients being female as opposed to 23.9% of patients being male. 45.4% of patients weighed between 66 and 86 kg, and 11.2% of patients weighed between 87 and 107 kg. A good number of the patients (73%) are self-employed, as shown by their occupational status.

**Table 1:** Demographic characteristics of type 2 diabetes patients

Category	Frequency	Percent
Age		
25–34	3	1.5
35–44	25	12.2
45–54	53	25.9
55–64	39	19.0
65–74	59	28.8
75–84	22	10.7
85–94	4	2.0
Total	205	100.0
Weight		
45–65	84	41.0
66–86	93	45.4
87–107	23	11.2
108–128	5	2.4
Total	205	100.0
Gender		
Male	49	23.9
Female	156	76.1
Total	205	100.0
Occupation		
Unemployed	10	4.9
Self-employed	149	73.0
Public or private sector employee	23	11.3
Pensioner	22	10.8
Total	204	100.0
Education		
None	115	56.1
Primary	23	11.2
JHS	5	2.4
SHS	4	2.0
Tertiary	58	28.3
Total	205	100.0

Table 2 shows that the 55–64 age group accounts for the largest proportion of hypertension patients, accounting for 31.4% of the total population of Patients with Hypertension. Few (15.7%) of the patients have age between 45 and 54. Among the patients who have hypertension, the weight distribution shows that the biggest proportion, which accounts for 50.2% of the total, falls within the range of 70–90 kg, followed by the range of 46–69 kg, which accounts for 41.3% of the total. This demonstrates that individuals with a normal weight can also be impacted by hypertension, despite the fact that being overweight or obese is associated with an increased risk of having the condition at some point in their lives. Based on the gender distribution, males have a higher (67.7%) hypertension compared to females (32.3%). According to the occupational status of patients, the highest proportion of them are self-employed, which accounts for 67.7% of the total. Public or private sector employees make up 19.3% of the total, and retirees account for 9.4% of the total. Patients’ educational backgrounds reveal that 49.8%of them have not gotten any kind of formal education, while 32.7% have completed their studies at a university level.

**Table 2:** Demographic characteristics of Hypertension patients

Category	Frequency	Percentage
Age		
25–34	29	13.0
35–44	22	9.9
45–54	35	15.7
55–64	70	31.4
65–74	42	18.8
75–84	25	11.2
Total	223	100.0
Weight		
46–69	92	41.3
70–90	112	50.2
91–111	14	6.3
112–132	5	2.2
Total	223	100.0
Gender		
Male	151	67.7
Female	72	32.3
Total	223	100.0
Occupation		
Unemployed	8	3.6
Self-employed	151	67.7
Public or private sector employee	43	19.3
Pensioner	21	9.4
Total	223	100.0
Education		
None	111	49.8
Primary	21	9.4
JHS	8	3.6
SHS	10	4.5
Tertiary	73	32.7
Total	223	100.0

The results in Table 3 above indicate significant variations in the prevalence of hypertension among patients across different demographic categories, such as age, weight, gender, occupation, and education level. The age group with the highest prevalence is 55–64 years, accounting for 31.4%. Significantly, all age groups exhibit p-values that are statistically significant (0.0001), providing substantial evidence against the null hypothesis that there is no association between age and the prevalence of hypertension. The weight categories also demonstrate a clear association, with the highest prevalence in the 70–90 kg range at 50.2% (though not statistically significant since p = 0.050) and a notable 41.3% in the 46–69 kg range. In terms of gender, the prevalence is markedly higher among males (67.7%) compared to females (32.3%), with both proportions showing strong statistical significance. The occupational status further highlights disparities, with those in the self-employed category exhibiting a prevalence of 67.7%. The prevalence among unemployed individuals is significantly lower, at 3.6%. Educational attainment also impacts hypertension prevalence, with those having no formal education showing a prevalence of 49.8%

**Table 3:** Prevalence of Hypertension among patients

Category	Proportion	Confidence Interval (95%)	P-value
Age 25–34	0.130	(0.086, 0.174)	0.0001
Age 35–44	0.099	(0.060, 0.138)	0.0001
Age 45–54	0.157	(0.112, 0.202)	0.0001
Age 55–64	0.314	(0.263, 0.365)	0.0001
Age 65–74	0.188	(0.141, 0.235)	0.0001
Age 75–84	0.112	(0.073, 0.151)	0.0001
Weight 46–69	0.413	(0.363, 0.463)	0.0001
Weight 70–90	0.502	(0.455, 0.549)	0.50
Weight 91–111	0.063	(0.034, 0.092)	0.0001
Weight 112–132	0.022	(0.004, 0.040)	0.0001
Male	0.678	(0.634, 0.722)	0.0001
Female	0.323	(0.278, 0.368)	0.0001
Unemployed	0.036	(0.016, 0.056)	0.0001
Self-employed	0.678	(0.634, 0.722)	0.0001
Public or private sector employee	0.193	(0.143, 0.243)	0.0001
Pensioner	0.094	(0.057, 0.131)	0.0001
None	0.498	(0.448, 0.548)	0.50
Primary	0.094	(0.057, 0.131)	0.0001
JHS	0.036	(0.016, 0.056)	0.0001
SHS	0.045	(0.020, 0.070)	0.0001
Tertiary	0.327	(0.277, 0.377)	0.0001

Results from Table 4 indicate that, the prevalence of Type 2 diabetes noticeably rises with age, peaking at 28.8% in the 65–74 age range. On the other hand, the age group of 25–34 has the lowest prevalence at 1.5%. The weight category follows a similar pattern, with the 45–65 age group showing the highest prevalence (41.0%). Nevertheless, a p-value of 0.500 suggests that the 66–86 category does not produce statistically significant results. There are clear disparities in prevalence between the sexes, with females showing a substantially higher prevalence (76.1%) than males (23.9%). All age, gender, and occupation groups show statistically significant p-values (all less than 0.0001), according to the results in Table 4. When considering occupation, it is observed that those who are self-employed have the highest prevalence at 72.7%, whereas the unemployed exhibit the lowest prevalence at 4.9%. The role of education level is also critical, as individuals with no formal education exhibit a prevalence rate of 56.1% Table 5.

**Table 4:** Prevalence of Type 2 diabetes among patients

Category	Subcategory	Frequency	Confidence Interval (95%)	P-value
Age	25–34	3	(0.000, 0.031)	0.0001
	35–44	25	(0.078, 0.166)	0.0001
	45–54	53	(0.203, 0.315)	0.0001
	55–64	39	(0.139, 0.241)	0.0001
	65–74	59	(0.230, 0.346)	0.0001
	75–84	22	(0.067, 0.147)	0.0001
	85–94	4	(0.001, 0.039)	0.0001
Weight	45–65	84	(0.344, 0.476)	0.0001
	66–86	93	(0.387, 0.521)	0.500
	87–107	23	(0.073, 0.151)	0.0001
	108–128	5	(0.004, 0.044)	0.0001
Gender	Male	49	(0.184, 0.294)	0.0001
	Female	156	(0.706, 0.816)	0.0001
Occupation	Unemployed	10	(0.021, 0.077)	0.0001
	Self-employed	149	(0.671, 0.789)	0.0001
	Public or private sector employee	23	(0.073, 0.153)	0.0001
	Pensioner	22	(0.068, 0.148)	0.0001
Education	None	115	(0.494, 0.628)	0.500
	Primary	23	(0.073, 0.151)	0.0001
	JHS	5	(0.004, 0.044)	0.0001
	SHS	4	(0.001, 0.039)	0.0001
	Tertiary	58	(0.225, 0.341)	0.0001

**Table 5:** Analysis of Variance (ANOVA)

Source	SS	df	MS
Model	964.839024	6	160.806504
Residual	643.226016	116	5.54419066
Total	1608.06504	122	13.180861

**Table:** 

Number of obs = 205
F(6, 116) = 2.23
Prob > F = 0.0452
R-squared = 0.600
Adj R-squared = 0.579

At the 5% level of significance, the whole regression model is statistically significant (F-statistic = 2.23, p = 0.0452). The dependent variable is strongly related to one or more of the model's predictors. The model successfully accounts for approximately 60.0% of the variation in the dependent variable, as indicated by the R-squared score of 0.600.

The regression results in Table 6 examine the relationship between socio-economic variables and the burden of Type 2 diabetes. The results indicate that gender and patients’ place of residence have a significant impact on glucose levels, while age, weight, occupation, and education do not have a notable effect. More precisely, the gender coefficient indicates that males have glucose levels that are 1.418 units greater than females, while keeping all other factors constant. The statistical analysis reveals a significant association (p = 0.046) between gender and glucose levels, indicating that gender plays a crucial role in determining glucose levels. Similarly, the coefficient for patients’ place of residence signifies that a one-unit shift in location results in a decrease of 0.028 units in glucose levels, providing all other factors remain constant. The result demonstrates a significant association (p = 0.030) between patients’ place of residence and glucose levels, suggesting that patients’ place of residence has a pivotal influence in determining glucose levels. However, the coefficients for age (−0.030, p = 0.237), weight (−0.018, p = 0.408), occupation (−0.052, p = 0.549), and education (0.064, p = 0.766) are not statistically significant. This indicates that these variables do not have a significant impact on glucose levels in this model. The constant term (11.513) indicates the mean glucose level when all other variables are set to zero.

**Table 6:** Regression analysis of the socio-economic factors contributing to the burden of Type 2 diabetes

Glucoselevel	Coef	Std. Err	t	P >|t|
Age	−0.030	0.025	−1.188	0.237
Weight	−0.018	0.022	−0.831	0.408
Gender	1.418	0.702	2.021	0.046
Occupation	−0.052	0.086	−0.602	0.549
Education	0.064	0.213	0.298	0.766
patients’ place	−0.028	0.013	−2.199	0.030
of residence				
_cons	11.513	3.003	3.834	0.000

**Table:** 

Number of obs = 223
F(5, 94) = 2.25
Prob > F = 0.0554
R-squared = 0.650
Adj R-squared = 0.631

Regression analysis as delineated in Table 7 evaluates the connection between many predictors and the dependent variable using 223 observations. At a 5% significance level, the F-statistic, which has a value of 2.25 and a p-value of 0.0554, indicates that the comprehensive model has a low level of significance. With an R-squared coefficient of 0.650, the independent variables in the model account for 65.0% of the variability in the dependent variable.

**Table 7:** Analysis of Variance (ANOVA)

Source	SS	df	MS
Model	2.3133546475	5	0.4626709295
Residual	1.2456525025	94	0.0132462979
Total	3.55900715	99	0.035949567

Regression analysis in Table 8 shows that many socioeconomic variables contribute to hypertension condition. The likelihood of hypertension increases with age, according to the positive and statistically significant age coefficient (Coef. = 0.0033, *p* = 0.015). Alternatively, this model does not find a statistically significant relationship between hypertension and weight, gender, occupation, or education (*p*-values > 0.05).

**Table 8:** Regression analysis of the socio-economic factors contributing to the burden of hypertension

BP	Coef	Std. Err	t	P >|t|	[95% Conf. Interval]	
Age	0.0032885	0.001322	2.49	0.015	0.0006636	0.0059134
Weight	−0.0011479	0.0013067	−0.88	0.382	−0.0037424	0.0014467
Gender	−0.0321872	0.0408754	−0.79	0.433	−0.1133462	0.0489718
Occupation	−0.0048411	0.0035501	−1.36	0.176	−0.0118898	0.0022077
Education	0.0097275	0.0119821	0.81	0.419	−0.0140632	0.0335182
_cons	1.67451	0.1637545	10.23	0.000	1.349372	1.999649

Socioeconomic status is one of the risk factors for developing hypertension and type 2 diabetes, according to multivariate multiple regression analysis. From Table 9 above, it appears that occupation of patients is linked to lower glucose levels, since the occupation coefficient is statistically significant (Coef. = −0.1478, p = 0.040) in relation to glucose levels. There is no strong influence of age, weight, gender, or education on glucose levels in this model, since the corresponding coefficients are not statistically significant (*p*-values > 0.05). When all other variables are set to zero, the baseline glucose level is represented by the constant term (_cons = 11.8745, *p* < 0.0001). The age coefficient in the blood pressure (BP) model is positive and statistically significant (Coef. = 0.0033, *p* = 0.015), which is in line with the preceding regression study. This finding further supports the idea that getting older is a major contributor to the development of hypertension. This model does not find a statistically significant relationship between blood pressure and gender, weight, employment, or level of education (*p*-values > 0.05). When all other variables are set to zero, the baseline blood pressure is represented by the constant term (_cons = 1.6745, *p* < 0.0001).

**Table 9:** Multivariate Multiple Regression analysis of the socio-economic factors contributing to the burden of hypertension and type 2 diabetes

	Coef	Std. Err	**t**	P >|t|	[95% Conf. Interval]	
Glucose level						
Age	-.0355033	.0263667	−1.35	0.181	-.0878549	.0168484
Weight	.0063572	.0260619	0.24	0.808	-.0453892	.0581036
Gender	.6590786	.8152323	0.81	0.421	-.9595842	2.277741
Occupation	1,478,288	.070804	−2.09	0.040	-.2884117	0072458
Education	.2397521	.2389746	1.00	0.318	-.2347375	. 7,142,417
cons	11.87446	3.265977	3.64	0.000	5.389787	18.35913
BP						
Age	0032885	.001322	2.49	0.015	.0006636	.0059134
Weight	0011479	.0013067	−0.88	−0.88	-.0037424	.0014467
Gender -	.0321872	.0408754	−0.79	0.433	-.1133462	.0489718
Occupation -	.0048411	.0035501	1.36	0.176	-.0118898	.0022077
Education	.0097275	.0119821	0.81	0.419	-.0140632	.0335182_
Cons	1.67451	.1637545	10.23	0.000	1.349372	1.999649

**Fig. 2:**
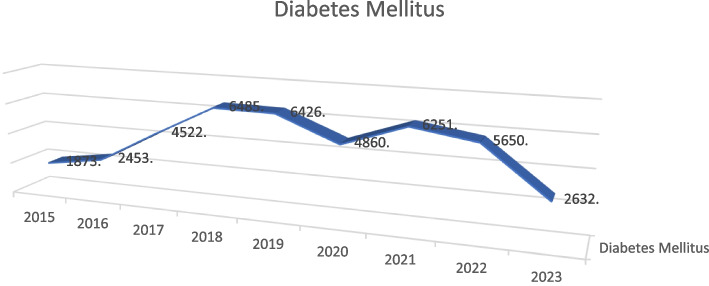
Trend analysis of Type 2 diabetes cases

Figure 2 reveals a general increase in diabetes mellitus cases from 2015 to 2018, reaching a peak in 2018, followed by fluctuations and a significant drop in 2023. The decomposition of the series indicates that the trend component shows a rising trend until 2018, after which it decreases. The seasonal component is minimal, indicating that the data does not exhibit strong seasonal patterns. The residuals suggest variations that are not explained by the trend or seasonal components. A linear trend graph indicates an average annual increase of approximately 241 cases per year over the given period. This suggests a consistent increase in Type 2 diabetes cases from 2015 to 2018, potentially due to factors that contributed to the rise in cases during these years. The peak in 2018 and subsequent fluctuations suggest potential interventions, changes in reporting practices, or other factors impacting the number of reported cases. The significant drop in 2023 indicates a major shift, possibly due to changes in healthcare policies, better disease management, or other socio-economic factors.

**Fig. 3:**
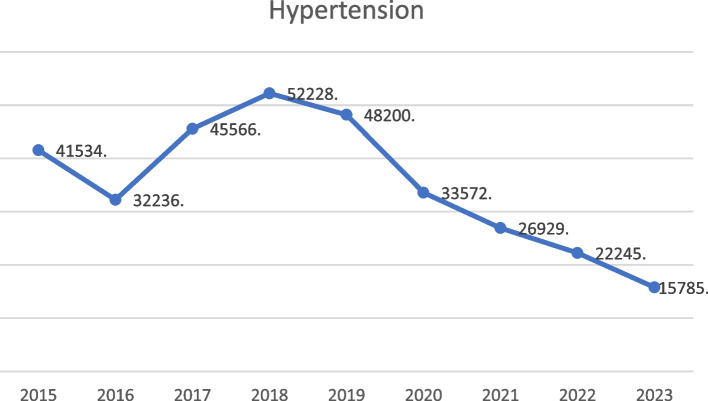
Trend analysis of hypertension cases

Figure 3 indicate a significant increase in hypertension cases from 2015 to 2018, rising from 41,534 to 52,228, which reflects an approximate increase of 25.8% and highlights a growing concern regarding hypertension in the population. Nonetheless, subsequent to attaining its zenith in 2018, a significant decrease in incidences is observed in the subsequent years. In the year 2019, there was a minor reduction to 48,200 cases, which was followed by a more noticeable decline in the years that followed: 33,572 in 2020, 26,929 in 2021, 22,245 in 2022, and ultimately 15,785 in 2023.

## Discussion

This study highlights the increasing prevalence of hypertension and type 2 diabetes among the elderly population in the Tamale metropolitan area, particularly among those aged 55–74 years. The significant association between aging and these diseases aligns with global evidence, which indicates that age is a major unmodifiable risk factor for chronic noncommunicable diseases [39],Cowie et al., 2018). This study deepens our understanding of diabetes prevalence in urban communities in Ghana and highlights that limitations in preventive treatment may accelerate disease progression among the elderly.

An important observation is the significant gender disparity in the incidence of type 2 diabetes, with women accounting for 76.1% of cases. While some global studies indicate higher incidence rates among men [19], regional factors such as social roles, caregiving responsibilities, dietary habits, and access to healthcare may explain why women have a higher prevalence in this context. This highlights the need for gender-responsive intervention measures, including specialized health education for women, community screening measures integrated into maternal and child health services, and strategies to address barriers to lifestyle changes.

Hypertension is most common among those aged 55–64, indicating that middle age is a critical stage for intervention. This aligns with the findings of Fleg [31] and Song et al. [75], who observed that blood pressure typically increases with age, with notable gender differences in older populations. While weight is clearly associated with hypertension and diabetes, not all weight categories yield statistically significant results, highlighting the complexity of using BMI as a predictive variable in resource-limited settings where both undernutrition and overnutrition occur.

The observed co-occurrence of hypertension and type 2 diabetes underscores the necessity of integrated non-communicable disease screening and management regimens at primary healthcare settings. Health services in areas such as Tamale must adjust to the dual burden by enhancing surveillance, facilitating early detection, and integrating culturally pertinent behavioral therapies.

## Conclusion

The study concludes that type 2 diabetes is most prevalent among older individuals, particularly those aged 65–74 years (28.8%), with the lowest prevalence observed among those aged 25–34 years (1.5%). The condition is significantly more common in females (76.1%) than in males (23.9%). In terms of weight, the highest proportion of diabetes cases occurred among individuals weighing 66–86 kg (45.4%). Self-employment was strongly associated with the condition, with 73.0% of diabetes patients engaged in self-employment, while only 4.9% were unemployed. Over half of the diabetes patients (56.1%) had no formal education, underscoring the influence of lower educational attainment.

Hypertension was most common among individuals aged 55–64 years (31.4%) and those within the 70–90 kg weight range (50.2%). Unlike diabetes, hypertension was more prevalent in males (67.7%) than females (32.3%). A significant majority of hypertension patients (67.7%) were also in self-employment, while 49.8% had no formal education, highlighting a strong link with socioeconomic disadvantage.

The study also found that gender and residence significantly influence glucose levels related to type 2 diabetes, whereas age, weight, occupation, and education did not show statistically significant effects. In contrast, age was the only socioeconomic factor with a statistically significant positive association with hypertension (p = 0.015), indicating that the risk of hypertension increases with advancing age.

The cross-sectional study design used in this study focused on collecting exposure and outcome data at a single point in time, offering a valuable snapshot of disease prevalence. However, their ability to establish causality or determine temporal relationships is limited, and they may be susceptible to selection bias and confounding. To enhance external validity and generalizability, future research should include multiple study sites, incorporating rural and peri-urban settings across Ghana. This approach would yield a more representative sample and facilitate comparisons across diverse populations.

## Data Availability

The data supporting the findings of this study are available from the corresponding author upon reasonable request.

## References

[1] Addo J, Agyemang C, de-Graft Aikins A, Beune E, Schulze MB, Danquah I, Galbete C, Nicolaou M, Meeks K, Klipstein-Grobusch K, Bahendaka S, Mockenhaupt FP, Owusu-Dabo E, Kunst A, Stronks K, Smeeth L. Association between socioeconomic position and the prevalence of type 2 diabetes in Ghanaians in different geographic locations: the RODAM study. J Epidemiol Community Health. 2017;71(7):633–9. 10.1136/jech-2016-208322. 28348205 10.1136/jech-2016-208322 PMC5485755

[2] Addo J, Smeeth L, Leon DA. Hypertension in sub-saharan Africa: a systematic review. Hypertension (Dallas, Tex: 1979). 2007;50(6):1012–8. 10.1161/HYPERTENSIONAHA.107.093336. 17954720 10.1161/HYPERTENSIONAHA.107.093336

[3] Adeloye D, Basquill C. Estimating the prevalence and awareness rates of hypertension in Africa: a systematic analysis. PLoS One. 2014;9(8): e104300. 10.1371/journal.pone.0104300. 25090232 10.1371/journal.pone.0104300 PMC4121276

[4] Adua E, Kolog EA, Afrifa-Yamoah E, Amankwah B, Obirikorang C, Anto EO, Acheampong E, Wang W, Tetteh AY. Predictive model and feature importance for early detection of type II diabetes mellitus. Transl Med Commun. 2021;6(1): 17. 10.1186/s41231-021-00096-z.

[5] Agardh E, Ahlbom A, Andersson T, Efendic S, Grill V, Hallqvist J, Ostenson C. Socio-economic position at three points in life in association with type 2 diabetes and impaired glucose tolerance in middle-aged Swedish men and women. Int J Epidemiol. 2007;36(1):84–92. 10.1093/ije/dyl269. 17510076 10.1093/ije/dyl269

[6] Agardh E, Allebeck P, Hallqvist J, Moradi T, Sidorchuk A. Type 2 diabetes incidence and socio-economic position: a systematic review and meta-analysis. Int J Epidemiol. 2011;40(3):804–18. 10.1093/ije/dyr029. 21335614 10.1093/ije/dyr029

[7] American Diabetes Association. Standards of Medical Care in Diabetes–2006. Diabetes Care. 2006; 29(suppl_1), s4–s42. 10.2337/diacare.29.s1.06.s4 16373931

[8] American Diabetes Association. Classification and Diagnosis of Diabetes: Standards of Medical Care in Diabetes-2019. Diabetes Care. 2019;42(Suppl 1):S13–28. 10.2337/dc19-S002. 30559228 10.2337/dc19-S002

[9] Asamoah-Boaheng M, Sarfo-Kantanka O, Tuffour AB, Eghan B, Mbanya JC. Prevalence and risk factors for diabetes mellitus among adults in Ghana: a systematic review and meta-analysis. Int Health. 2019;11(2):83–92. 10.1093/inthealth/ihy067. 30285118 10.1093/inthealth/ihy067

[10] Aune D, Sen A, Schlesinger S, Norat T, Janszky I, Romundstad P, Tonstad S, Riboli E, Vatten LJ. Body mass index, abdominal fatness, fat mass and the risk of atrial fibrillation: a systematic review and dose-response meta-analysis of prospective studies. Eur J Epidemiol. 2017;32(3):181–92. 10.1007/s10654-017-0232-4. 28194602 10.1007/s10654-017-0232-4 PMC5380695

[11] Bayaraa, N., Azahar, N. M., Kitaoka, K., Kobayashi, Y., & Yano, Y. African Control of Hypertension through Innovative Epidemiology and a Vibrant Ecosystem (ACHIEVE): A holistic approach for hypertension control in Africa. Journal of Human Hypertension. 2023; s41371–023–00845–00847. 10.1038/s41371-023-00845-7 10.1038/s41371-023-00845-7 37386123

[12] Bitew ZW, Alemu A, Jember DA, Tadesse E, Getaneh FB, Sied A, Weldeyonnes M. Prevalence of glycemic control and factors associated with poor glycemic control: a systematic review and meta-analysis. Inquiry. 2023;60: 469580231155716. 10.1177/00469580231155716. 36852627 10.1177/00469580231155716 PMC10071101

[13] Bommer C, Sagalova V, Heesemann E, Manne-Goehler J, Atun R, Bärnighausen T, Davies J, Vollmer S. Global economic burden of diabetes in adults: projections from 2015 to 2030. Diabetes Care. 2018;41(5):963–70. 10.2337/dc17-1962. 29475843 10.2337/dc17-1962

[14] Bosu WK, Bosu DK. Prevalence, awareness and control of hypertension in Ghana: a systematic review and meta-analysis. PLoS One. 2021;16(3): e0248137. 10.1371/journal.pone.0248137. 33667277 10.1371/journal.pone.0248137 PMC7935309

[15] Brook RD, Rajagopalan S, Pope CA, Brook JR, Bhatnagar A, Diez-Roux AV, Holguin F, Hong Y, Luepker RV, Mittleman MA, Peters A, Siscovick D, Smith SC, Whitsel L, Kaufman JD. Particulate matter air pollution and cardiovascular disease: an update to the scientific statement from the American Heart Association. Circulation. 2010;121(21):2331–78. 10.1161/CIR.0b013e3181dbece1. 20458016 10.1161/CIR.0b013e3181dbece1

[16] Brownson RC, Fielding JE, Maylahn CM. Evidence-based public health: a fundamental concept for public health practice. Annu Rev Public Health. 2009;30:175–201. 10.1146/annurev.publhealth.031308.100134. 19296775 10.1146/annurev.publhealth.031308.100134

[17] Carretero OA, Oparil S. Essential hypertension: part I: definition and etiology. Circulation. 2000;101(3):329–35. 10.1161/01.CIR.101.3.329. 10645931 10.1161/01.cir.101.3.329

[18] Chatterjee S, Davies MJ, Heller S, Speight J, Snoek FJ, Khunti K. Diabetes structured self-management education programmes: a narrative review and current innovations. Lancet Diabetes Endocrinol. 2018;6(2):130–42. 10.1016/S2213-8587(17)30239-5. 28970034 10.1016/S2213-8587(17)30239-5

[19] Cheng YJ, Imperatore G, Geiss LS, Wang J, Saydah SH, Cowie CC, Gregg EW. Secular changes in the age-specific prevalence of diabetes among U.S. adults: 1988–2010. Diabetes Care. 2013;36(9):2690–6. 10.2337/dc12-2074. 23637354 10.2337/dc12-2074 PMC3747941

[20] Cho NH, Shaw JE, Karuranga S, Huang Y, da Rocha Fernandes JD, Ohlrogge AW, Malanda B. IDF diabetes atlas: global estimates of diabetes prevalence for 2017 and projections for 2045. Diabetes Res Clin Pract. 2018;138:271–81. 10.1016/j.diabres.2018.02.023. 29496507 10.1016/j.diabres.2018.02.023

[21] Chobanian AV, Bakris GL, Black HR, Cushman WC, Green LA, Izzo JL, Jones DW, Materson BJ, Oparil S, Wright JT, Roccella EJ, the National High Blood Pressure Education Program Coordinating Committee. Seventh report of the joint national committee on prevention, detection, evaluation, and treatment of high blood pressure. Hypertension. 2003;42(6):1206–52. 10.1161/01.HYP.0000107251.49515.c2. 14656957 10.1161/01.HYP.0000107251.49515.c2

[22] Colagiuri S, Borch-Johnsen K, Glümer C, Vistisen D. There really is an epidemic of type 2 diabetes. Diabetologia. 2005;48(8):1459–63. 10.1007/s00125-005-1843-y. 16007413 10.1007/s00125-005-1843-y

[23] Coster S, Gulliford MC, Seed PT, Powrie JK, Swaminathan R. Self-monitoring in type 2 diabetes mellitus: a meta-analysis. Diabet Med. 2000;17(11):755–61. 10.1046/j.1464-5491.2000.00390.x. 11131099 10.1046/j.1464-5491.2000.00390.x

[24] Cowie, C. C., Casagrande, S. S., & Geiss, L. S. Prevalence and Incidence of Type 2 Diabetes and Prediabetes. In C. C. Cowie, S. S. Casagrande, A. Menke, M. A. Cissell, M. S. Eberhardt, J. B. Meigs, E. W. Gregg, W. C. Knowler, E. Barrett-Connor, D. J. Becker, F. L. Brancati, E. J. Boyko, W. H. Herman, B. V. Howard, K. M. V. Narayan, M. Rewers, & J. E. Fradkin (Eds.), Diabetes in America (3rd ed.). 2018; National Institute of Diabetes and Digestive and Kidney Diseases (US). http://www.ncbi.nlm.nih.gov/books/NBK568004/33651562

[25] Dai B, Addai-Dansoh S, Nutakor JA, Osei-Kwakye J, Larnyo E, Oppong S, Boahemaa PY, Arboh F. The prevalence of hypertension and its associated risk factors among older adults in Ghana. Front Cardiovasc Med. 2022;9: 990616. 10.3389/fcvm.2022.990616. 36606290 10.3389/fcvm.2022.990616 PMC9807661

[26] Danquah I, Bedu-Addo G, Terpe K-J, Micah F, Amoako YA, Awuku YA, Dietz E, van der Giet M, Spranger J, Mockenhaupt FP. Diabetes mellitus type 2 in urban Ghana: characteristics and associated factors. BMC Public Health. 2012;12:210. 10.1186/1471-2458-12-210. 22429713 10.1186/1471-2458-12-210 PMC3364878

[27] Davies MJ, Aroda VR, Collins BS, Gabbay RA, Green J, Maruthur NM, Rosas SE, Del Prato S, Mathieu C, Mingrone G, Rossing P, Tankova T, Tsapas A, Buse JB. Management of hyperglycaemia in type 2 diabetes, 2022. a consensus report by the American Diabetes Association (ADA) and the European Association for the Study of Diabetes (EASD). Diabetologia. 2022;65(12):1925–66. 10.1007/s00125-022-05787-2. 36151309 10.1007/s00125-022-05787-2 PMC9510507

[28] Doherty ML, Owusu-Dabo E, Kantanka OS, Brawer RO, Plumb JD. Type 2 diabetes in a rapidly urbanizing region of Ghana, West Africa: a qualitative study of dietary preferences, knowledge and practices. BMC Public Health. 2014;14:1069. 10.1186/1471-2458-14-1069. 25312471 10.1186/1471-2458-14-1069 PMC4287448

[29] Escobales N, Crespo MJ. Oxidative-nitrosative stress in hypertension. Curr Vasc Pharmacol. 2005;3(3):231–46. 10.2174/1570161054368643. 16026320 10.2174/1570161054368643

[30] Ferdinand KC. Uncontrolled hypertension in sub-Saharan Africa: now is the time to address a looming crisis. J Clin Hypertens (Greenwich). 2020;22(11):2111–3. 10.1111/jch.14046. 32951284 10.1111/jch.14046 PMC8030060

[31] Fleg JL. Healthy lifestyle and risk of heart failure: an ounce of prevention well worth the effort. Circ Heart Fail. 2016;9(4): e003155. 10.1161/CIRCHEARTFAILURE.116.003155. 27072862 10.1161/CIRCHEARTFAILURE.116.003155 PMC4955672

[32] Franklin SS, Larson MG, Khan SA, Wong ND, Leip EP, Kannel WB, Levy D. Does the relation of blood pressure to coronary heart disease risk change with aging? The Framingham Heart Study. Circulation. 2001;103(9):1245–9. 10.1161/01.CIR.103.9.1245. 11238268 10.1161/01.cir.103.9.1245

[33] Gatimu SM, Milimo BW, Sebastian MS. Prevalence and determinants of diabetes among older adults in Ghana. BMC Public Health. 2016;16(1):1174. 10.1186/s12889-016-3845-8. 27871259 10.1186/s12889-016-3845-8 PMC5117548

[34] GBD 2015 Obesity Collaborators, Afshin, A., Forouzanfar, M. H., Reitsma, M. B., Sur, P., Estep, K., Lee, A., Marczak, L., Mokdad, A. H., Moradi-Lakeh, M., Naghavi, M., Salama, J. S., Vos, T., Abate, K. H., Abbafati, C., Ahmed, M. B., Al-Aly, Z., Alkerwi, A., Al-Raddadi, R., … Murray, C. J. L. Health Effects of Overweight and Obesity in 195 Countries over 25 Years. The New England Journal of Medicine. 2017; 377(1), 13–27. 10.1056/NEJMoa1614362 10.1056/NEJMoa1614362 PMC5477817 28604169

[35] Gelber RP, Gaziano JM, Manson JE, Buring JE, Sesso HD. A prospective study of body mass index and the risk of developing hypertension in men. Am J Hypertens. 2007;20(4):370–7. 10.1016/j.amjhyper.2006.10.011. 17386342 10.1016/j.amjhyper.2006.10.011 PMC1920107

[36] Ghana, G. S. S. Population and Housing Census: General Report. GSS: Accra, Ghana. 2021; 1–81. (n.d.).

[37] Gill GV, Mbanya J-C, Ramaiya KL, Tesfaye S. A sub-Saharan African perspective of diabetes. Diabetologia. 2009;52(1):8–16. 10.1007/s00125-008-1167-9. 18846363 10.1007/s00125-008-1167-9

[38] Goryakin Y, Rocco L, Suhrcke M. The contribution of urbanization to non-communicable diseases: evidence from 173 countries from 1980 to 2008. Econ Hum Biol. 2017;26:151–63. 10.1016/j.ehb.2017.03.004. 28410489 10.1016/j.ehb.2017.03.004

[39] Gregg EW, Zhuo X, Cheng YJ, Albright AL, Narayan KMV, Thompson TJ. Trends in lifetime risk and years of life lost due to diabetes in the USA, 1985–2011: a modelling study. Lancet Diabetes Endocrinol. 2014;2(11):867–74. 10.1016/S2213-8587(14)70161-5. 25128274 10.1016/S2213-8587(14)70161-5

[40] Grossman A, Messerli FH, Grossman E. Drug induced hypertension – an unappreciated cause of secondary hypertension. Eur J Pharmacol. 2015;763:15–22. 10.1016/j.ejphar.2015.06.027. 26096556 10.1016/j.ejphar.2015.06.027

[41] Gudjinu HY, Sarfo B. Risk factors for type 2 diabetes mellitus among out-patients in Ho, the Volta regional capital of Ghana: a case-control study. BMC Res Notes. 2017;10(1):324. 10.1186/s13104-017-2648-z. 28747218 10.1186/s13104-017-2648-z PMC5530523

[42] Guh DP, Zhang W, Bansback N, Amarsi Z, Birmingham CL, Anis AH. The incidence of co-morbidities related to obesity and overweight: a systematic review and meta-analysis. BMC Public Health. 2009;9:88. 10.1186/1471-2458-9-88. 19320986 10.1186/1471-2458-9-88 PMC2667420

[43] Hall V, Thomsen RW, Henriksen O, Lohse N. Diabetes in sub Saharan Africa 1999–2011: epidemiology and public health implications. A systematic review. BMC Public Health. 2011;11: 564. 10.1186/1471-2458-11-564. 21756350 10.1186/1471-2458-11-564 PMC3156766

[44] He S, Wang L, Miao L, Wang T, Du F, Zhao L, Wang X. Receptor interacting protein kinase-3 determines cellular necrotic response to TNF-α. Cell. 2009;137(6):1100–11. 10.1016/j.cell.2009.05.021. 19524512 10.1016/j.cell.2009.05.021

[45] Hilawe EH, Yatsuya H, Kawaguchi L, Aoyama A. Differences by sex in the prevalence of diabetes mellitus, impaired fasting glycaemia and impaired glucose tolerance in sub-Saharan Africa: a systematic review and meta-analysis. Bull World Health Organ. 2013;91(9):671-682D. 10.2471/BLT.12.113415. 24101783 10.2471/BLT.12.113415 PMC3790213

[46] Hollingworth SA, Ankrah D, Uzochukwu BSC, Okeke CC, Ruiz F, Thacher E. Antihypertensive medicine use differs between Ghana and Nigeria. BMC Cardiovasc Disord. 2022;22(1):368. 10.1186/s12872-022-02799-z. 35948937 10.1186/s12872-022-02799-z PMC9364553

[47] Holt-Lunstad J, Smith TB, Layton JB. Social relationships and mortality risk: a meta-analytic review. PLoS Med. 2010;7(7): e1000316. 10.1371/journal.pmed.1000316. 20668659 10.1371/journal.pmed.1000316 PMC2910600

[48] Hwang J, Shon C. Relationship between socioeconomic status and type 2 diabetes: results from Korea National Health and Nutrition Examination Survey (KNHANES) 2010–2012. BMJ Open. 2014;4(8): e005710. 10.1136/bmjopen-2014-005710. 10.1136/bmjopen-2014-005710 PMC4139629 25138810

[49] Kasper, D. L. (Ed.). Harrison’s principles of internal medicine (19th edition / editors, Dennis L. Kasper, MD, William Ellery Channing, Professor of Medicine, Professor of Microbiology, Department of Microbiology and Immunobiology, Harvard Medical School, Division of Infectious Diseases, Brigham and Women's Hospital, Boston, Massachusetts [and five others]). McGraw Hill Education. 2015

[50] Kautzky-Willer A, Harreiter J, Pacini G. Sex and gender differences in risk, pathophysiology and complications of type 2 diabetes mellitus. Endocr Rev. 2016;37(3):278–316. 10.1210/er.2015-1137. 27159875 10.1210/er.2015-1137 PMC4890267

[51] Kearney PM, Whelton M, Reynolds K, Muntner P, Whelton PK, He J. Global burden of hypertension: analysis of worldwide data. Lancet. 2005;365(9455):217–23. 10.1016/S0140-6736(05)17741-1. 15652604 10.1016/S0140-6736(05)17741-1

[52] Khunti K, Aroda VR, Aschner P, Chan JCN, Del Prato S, Hambling CE, Harris S, Lamptey R, McKee M, Tandon N, Valabhji J, Seidu S. The impact of the COVID-19 pandemic on diabetes services: planning for a global recovery. Lancet Diabetes Endocrinol. 2022;10(12):890–900. 10.1016/S2213-8587(22)00278-9. 36356612 10.1016/S2213-8587(22)00278-9 PMC9640202

[53] Kotsis V, Stabouli S, Papakatsika S, Rizos Z, Parati G. Mechanisms of obesity-induced hypertension. Hypertens Res. 2010;33(5):386–93. 10.1038/hr.2010.9. 20442753 10.1038/hr.2010.9

[54] Kushitor SB, Owusu L, Kushitor MK. The prevalence and correlates of the double burden of malnutrition among women in Ghana. PLoS One. 2020;15(12): e0244362. 10.1371/journal.pone.0244362. 33370352 10.1371/journal.pone.0244362 PMC7769247

[55] Landsbergis PA, Dobson M, Koutsouras G, Schnall P. Job strain and ambulatory blood pressure: a meta-analysis and systematic review. Am J Public Health. 2013;103(3):e61-71. 10.2105/AJPH.2012.301153. 10.2105/AJPH.2012.301153 PMC3673518 23327240

[56] Lee J-Y, Nagano Y, Taylor JP, Lim KL, Yao T-P. Disease-causing mutations in Parkin impair mitochondrial ubiquitination, aggregation, and HDAC6-dependent mitophagy. J Cell Biol. 2010;189(4):671–9. 10.1083/jcb.201001039. 20457763 10.1083/jcb.201001039 PMC2872903

[57] Leng B, Jin Y, Li G, Chen L, Jin N. Socioeconomic status and hypertension: a meta-analysis. J Hypertens. 2015;33(2):221–9. 10.1097/HJH.0000000000000428. 25479029 10.1097/HJH.0000000000000428

[58] Li J, Shi L, Li S, Xu L, Qin W, Wang H. Urban-rural disparities in hypertension prevalence, detection, and medication use among Chinese adults from 1993 to 2011. Int J Equity Health. 2017;16(1):50. 10.1186/s12939-017-0545-7. 28288635 10.1186/s12939-017-0545-7 PMC5348878

[59] Magliano, D. J., Boyko, E. J., & IDF Diabetes Atlas 10th edition scientific committee. IDF DIABETES ATLAS (10th ed.). International Diabetes Federation. 2021; http://www.ncbi.nlm.nih.gov/books/NBK581934/35914061

[60] Mayosi BM, Lawn JE, Van Niekerk A, Bradshaw D, Abdool Karim SS, Coovadia HM. Health in South Africa: changes and challenges since 2009. Lancet. 2012;380(9858):2029–43. 10.1016/S0140-6736(12)61814-5. 23201214 10.1016/S0140-6736(12)61814-5

[61] Mbanya JC, Kengne AP, Assah F. Diabetes care in Africa. Lancet. 2006;368(9548):1628–9. 10.1016/S0140-6736(06)69673-6. 17098063 10.1016/S0140-6736(06)69673-6

[62] Mensah GA. Descriptive epidemiology of cardiovascular risk factors and diabetes in sub-Saharan Africa. Prog Cardiovasc Dis. 2013;56(3):240–50. 10.1016/j.pcad.2013.10.014. 24267431 10.1016/j.pcad.2013.10.014 PMC11646150

[63] Mills KT, Bundy JD, Kelly TN, Reed JE, Kearney PM, Reynolds K, Chen J, He J. Global disparities of hypertension prevalence and control: a systematic analysis of population-based studies from 90 countries. Circulation. 2016;134(6):441–50. 10.1161/CIRCULATIONAHA.115.018912. 27502908 10.1161/CIRCULATIONAHA.115.018912 PMC4979614

[64] Mills KT, Stefanescu A, He J. The global epidemiology of hypertension. Nat Rev Nephrol. 2020;16(4):223–37. 10.1038/s41581-019-0244-2. 32024986 10.1038/s41581-019-0244-2 PMC7998524

[65] Minicuci N, Biritwum RB, Mensah G, Yawson AE, Naidoo N, Chatterji S, Kowal P. Sociodemographic and socioeconomic patterns of chronic non-communicable disease among the older adult population in Ghana. Glob Health Action. 2014;7:21292. 10.3402/gha.v7.21292. 24746141 10.3402/gha.v7.21292 PMC3991840

[66] Mwangome M, Ngari M, Fegan G, Mturi N, Shebe M, Bauni E, Berkley JA. Diagnostic criteria for severe acute malnutrition among infants aged under 6 mo. Am J Clin Nutr. 2017;105(6):1415–23. 10.3945/ajcn.116.149815. 28424189 10.3945/ajcn.116.149815 PMC5445677

[67] Natella F, Scaccini C. Role of coffee in modulation of diabetes risk. Nutr Rev. 2012;70(4):207–17. 10.1111/j.1753-4887.2012.00470.x. 22458694 10.1111/j.1753-4887.2012.00470.x

[68] Nguyen TT, Tchetgen Tchetgen EJ, Kawachi I, Gilman SE, Walter S, Glymour MM. Comparing alternative effect decomposition methods: the role of literacy in mediating educational effects on mortality. Epidemiology. 2016;27(5):670–6. 10.1097/EDE.0000000000000517. 27280331 10.1097/EDE.0000000000000517 PMC5051696

[69] Noncommunicable diseases: Mortality. (n.d.).

[70] Obirikorang Y, Obirikorang C, Odame Anto E, Acheampong E, Dzah N, Akosah CN, Nsenbah EB. Knowledge and lifestyle-associated prevalence of obesity among newly diagnosed type II diabetes mellitus patients attending diabetic clinic at Komfo Anokye Teaching Hospital, Kumasi, Ghana: a hospital-based cross-sectional study. J Diabetes Res. 2016. 10.1155/2016/9759241. 10.1155/2016/9759241 PMC4736955 26881262

[71] Ong SE, Koh JJK, Toh S-AES, Chia KS, Balabanova D, McKee M, Perel P, Legido-Quigley H. Assessing the influence of health systems on type 2 diabetes mellitus awareness, treatment, adherence, and control: a systematic review. PLoS One. 2018;13(3): e0195086. 10.1371/journal.pone.0195086. 29596495 10.1371/journal.pone.0195086 PMC5875848

[72] Roux AVD, Merkin SS, Arnett D, Chambless L, Massing M, Nieto FJ, Sorlie P, Szklo M, Tyroler HA, Watson RL. Neighborhood of residence and incidence of coronary heart disease. N Engl J Med. 2001;345(2):99–106. 10.1056/NEJM200107123450205. 11450679 10.1056/NEJM200107123450205

[73] Seeman T, Palyzová D, Dusek J, Janda J. Reduced nocturnal blood pressure dip and sustained nighttime hypertension are specific markers of secondary hypertension. J Pediatr. 2005;147(3):366–71. 10.1016/j.jpeds.2005.04.042. 16182677 10.1016/j.jpeds.2005.04.042

[74] Shaw JE, Sicree RA, Zimmet PZ. Global estimates of the prevalence of diabetes for 2010 and 2030. Diabetes Res Clin Pract. 2010;87(1):4–14. 10.1016/j.diabres.2009.10.007. 19896746 10.1016/j.diabres.2009.10.007

[75] Song J-J, Ma Z, Wang J, Chen L-X, Zhong J-C. Gender differences in hypertension. J Cardiovasc Transl Res. 2020;13(1):47–54. 10.1007/s12265-019-09888-z. 31044374 10.1007/s12265-019-09888-z

[76] Spinaci M, Volpe S, Bernardini C, De Ambrogi M, Tamanini C, Seren E, Galeati G. Sperm sorting procedure induces a redistribution of Hsp70 but not Hsp60 and Hsp90 in boar spermatozoa. J Androl. 2006;27(6):899–907. 10.2164/jandrol.106.001008. 16870948 10.2164/jandrol.106.001008

[77] Stein, D. J., Aguilar-Gaxiola, S., Alonso, J., Bruffaerts, R., de Jonge, P., Liu, Z., Miguel Caldas-de-Almeida, J., O’Neill, S., Viana, M. C., Al-Hamzawi, A. O., Angermeyer, M. C., Benjet, C., de Graaf, R., Ferry, F., Kovess-Masfety, V., Levinson, D., de Girolamo, G., Florescu, S., Hu, C., … Scott, K. M. Associations between mental disorders and subsequent onset of hypertension. General Hospital Psychiatry. 2014; 36(2), 142–149. 10.1016/j.genhosppsych.2013.11.002 10.1016/j.genhosppsych.2013.11.002 PMC3996437 24342112

[78] Stringhini S, Tabak AG, Akbaraly TN, Sabia S, Shipley MJ, Marmot MG, Brunner EJ, Batty GD, Bovet P, Kivimäki M. Contribution of modifiable risk factors to social inequalities in type 2 diabetes: prospective Whitehall II cohort study. BMJ. 2012;345: e5452. 10.1136/bmj.e5452. 22915665 10.1136/bmj.e5452 PMC3424226

[79] Tannor EK, Nyarko OO, Adu-Boakye Y, Owusu Konadu S, Opoku G, Ankobea-Kokroe F, Opare-Addo M, Appiah LT, Amuzu EX, Ansah GJ, Appiah-Boateng K, Ofori E, Ansong D. Prevalence of hypertension in Ghana: analysis of an awareness and screening campaign in 2019. Clin Med Insights Cardiol. 2022;16:117954682211200. 10.1177/11795468221120092. 10.1177/11795468221120092 PMC9434666 36060113

[80] Tesfa E, Demeke D. Prevalence of and risk factors for hypertension in Ethiopia: a systematic review and meta-analysis. Health Sci Rep. 2021;4(3): e372. 10.1002/hsr2.372. 34589614 10.1002/hsr2.372 PMC8459032

[81] Tol A, Sharifirad G, Shojaezadeh D, Tavasoli E, Azadbakht L. Socio-economic factors and diabetes consequences among patients with type 2 diabetes. J Educ Health Promot. 2013;2: 12. 10.4103/2277-9531.108009. 24083262 10.4103/2277-9531.108009 PMC3778578

[82] Vialle-Valentin CE, Serumaga B, Wagner AK, Ross-Degnan D. Evidence on access to medicines for chronic diseases from household surveys in five low- and middle-income countries. Health Policy Plann. 2015;30(8):1044–52. 10.1093/heapol/czu107. 10.1093/heapol/czu107 PMC4654757 25255920

[83] Wang L, Wong TY, Sharrett AR, Klein R, Folsom AR, Jerosch-Herold M. Relationship between retinal arteriolar narrowing and myocardial perfusion: multi-ethnic study of atherosclerosis. Hypertension. 2008;51(1):119–26. 10.1161/HYPERTENSIONAHA.107.098343. 17998474 10.1161/HYPERTENSIONAHA.107.098343

[84] Wareham NJ, Forouhi NG. Is there really an epidemic of diabetes? Diabetologia. 2005;48(8):1454–5. 10.1007/s00125-005-1845-9. 16001234 10.1007/s00125-005-1845-9

[85] Werfalli M, Engel ME, Musekiwa A, Kengne AP, Levitt NS. The prevalence of type 2 diabetes among older people in Africa: a systematic review. Lancet Diabetes Endocrinol. 2016;4(1):72–84. 10.1016/S2213-8587(15)00363-0. 26548379 10.1016/S2213-8587(15)00363-0

[86] Whelton PK, He J, Appel LJ, Cutler JA, Havas S, Kotchen TA, Roccella EJ, Stout R, Vallbona C, Winston MC, Karimbakas J, National High Blood Pressure Education Program Coordinating Committee. Primary prevention of hypertension: clinical and public health advisory from the National High Blood Pressure Education Program. JAMA. 2002;288(15):1882–8. 10.1001/jama.288.15.1882. 12377087 10.1001/jama.288.15.1882

[87] Whitton SW, Whisman MA. Relationship satisfaction instability and depression. J Fam Psychol. 2010;24(6):791–4. 10.1037/a0021734. 21171780 10.1037/a0021734 PMC3535225

[88] Wild S, Roglic G, Green A, Sicree R, King H. Global prevalence of diabetes: estimates for the year 2000 and projections for 2030. Diabetes Care. 2004;27(5):1047–53. 10.2337/diacare.27.5.1047. 15111519 10.2337/diacare.27.5.1047

[89] Yeh ETH, Bickford CL. Cardiovascular complications of cancer therapy. J Am Coll Cardiol. 2009;53(24):2231–47. 10.1016/j.jacc.2009.02.050. 19520246 10.1016/j.jacc.2009.02.050

[90] Zeru MA, Tesfa E, Mitiku AA, Seyoum A, Bokoro TA. Prevalence and risk factors of type-2 diabetes mellitus in Ethiopia: systematic review and meta-analysis. Sci Rep. 2021;11(1):21733. 10.1038/s41598-021-01256-9. 34741064 10.1038/s41598-021-01256-9 PMC8571297

[91] Zimmet PZ. Diabetes and its drivers: the largest epidemic in human history? Clin Diabetes Endocrinol. 2017;3:1. 10.1186/s40842-016-0039-3. 28702255 10.1186/s40842-016-0039-3 PMC5471716

